# Cases of the Fatal Disease Which Occurred, during the Last Autumn, in His Majesty's Dock-Yard at Devonport; with Observations

**Published:** 1825-09

**Authors:** E. Tripe

**Affiliations:** Surgeon, Devonport, &c. &c.


THE LONDON
### Medical and Physical Journal.

No 3 OF VOL. LIV.] SEPTEMBER, 1825. [No 319.

For many fortunate discoveries in medicine, and for the detection of numerous errors, the world is indebted to the rapid circulation of Monthly Journals; and there never existed any work, to which the Faculty, in Europe and America, were under deeper obligations, than to the Medical and Physical Journal of London, now forming a long, but an invaluable, series.—RUSH.

### ORIGINAL COMMUNICATIONS,

### SELECT OBSERVATIONS, &c.

ART. I.—*Cases of the Fatal Disease which occurred, during the last Autumn, in His Majesty's Dock-yard at Devonport; with Observations.* By F. TRIPE, Esq. Surgeon, Devonport, &c. &c.

RICHARD QUICK, a smith, of his Majesty's dock-yard, aged thirty-eight, a man of regular, temperate habits, and cheerful disposition, had been for two years a subject of chronic visceral disease, but had latterly enjoyed a robust state of health. He returned from his duty on the evening of the 21st of September last, much indisposed; and the next morning he took a saline purgative, and absented himself from his labour. His indisposition continued to increase, and on the morning of the 23d I was requested to visit him. His face was much flushed; skin hot; pulse quick and full; tongue moist, and covered with a whitish coat. He complained of excessive pain and throbbing of his head, with weight in the occiput, and of great pain and tenderness in the right hypochondrium. The conjunctivæ were suffused, and all his movements and expressions were indicative of high cerebral irritation. His bowels had been effectively acted upon by the salts which he took the day before.

He was immediately bled until the force of the circulation was subdued, and an approach to faintness was indicated; when his feelings were generally relieved, and the distress and pain of his head entirely removed. He was directed to take calomel, with tartarised antimony, every six hours, and a solution of sulphate of magnesia.

24th.—No return of pain or throbbing of the head since yesterday; heat of skin decreased; pulse greatly reduced in frequency and force; pain and tenderness of hypochondrium also relieved. Considers himself very much better, in every particular, than on the preceding day. Yet, notwithstanding this general improvement, there still exists some evidences of cerebral irritation: his mind appears to be more than commonly susceptible of impressions, and, in communicating them, he expresses himself with unusual quickness. Medicines have acted well; the excretions are very dark and fetid.

Infusion of senna, with sulphate of magnesia, to be taken directly; and the pills to be continued, with a mixture containing the liquor ammoniæ acetatis; a blister to be applied to the nape of the neck.

25th.—Has refused to apply the blister, or to take his medicines. Has been in the strongest irritation of mind, with almost every impression delusive; his conduct was occasionally violent, and he exclaimed against me in particular, (for whom he had always, during a long professional acquaintance, appeared to entertain the warmest respect,) and charged me with having intentionally omitted that treatment which had proved so beneficial on the first day of my attendance; adding, that the pain had been thereby removed into both his legs, in which he at this time suffered considerable agony. He vehemently declared that he would submit to no further treatment. To repeated inquiries, he answered that he had no pain of his head or side. The skin and tongue did not exhibit any increase of morbid appearance, but the pulse was advanced in frequency and fulness. No alvine discharges.

A red patch, about the circumference of a crown-piece, has shown itself upon the outside of the calf of the right leg; and, on examining the limb, I find there has been a recent injury of the great toe, with the loss of the nail; but, on the most careful investigation, I cannot discover any unsoundness, or produce the least uneasiness by pressure. The entire line from the spot upon the calf to the toe is without increased heat, redness, tumefaction, or preternatural sensibility. The following history of the hurt was communicated to me:—A month had elapsed since the great toe was injured by a piece of iron falling upon it, while at work in the dock-yard, for which the surgeons of that establishment attended him; and, at the end of a fortnight, he returned to his employment, cured, and regularly followed his work until the 21st of September, when, it has already been stated, he returned to his home indisposed. In the course of the last day, while at his work, he frequently complained of uneasiness in his toe, and he had removed his stocking to examine it, when he pressed a little matter from one corner. This is the only evidence, however, which we have that he felt the least inconvenience in or near the original hurt, throughout any part of the case.

In the course of the afternoon (25th) he became tranquil, and his impressions quite correct: he recollected what had passed in the morning, and felt much distressed lest his conduct towards me (which he considered had been disrespectful,) might have offended me. He expressed the most earnest desire that I should retain no resentment; and he entreated that the efforts to relieve him might be renewed, making an exception only to the blister, the application of which he resisted in the most positive manner. He declares himself free from pain in his head and side, but describes the pain in the extremities to be most intense. There is no alteration in the appearance of the spot on the leg, the toe, or any intermediate part between them. —The purgative was ordered to be taken directly, and the pills and saline mixture as last directed; the toe to be enveloped in a bread-and-water cataplasm, and the legs to be bathed in the decoction of poppies.

26th.—Had some sleep, and altogether passed an easier night. Tongue coated as before; pulse undiminished in frequency and force; pain no where but in the legs, which continues equally severe; mind and general feelings nearly in the same condition as last evening. A second red patch has made its appearance upon the outside of the thigh. Medicines have acted well; the excretions continue very dark and fetid.—The medicines, cataplasm, and fomentations, were continued.

27th.—Perfectly incoherent; exhibits great irascibility, varied with levity, and occasional violence of expression and gesture. Does not complain of pain in any part, excepting in the right leg, which is swollen in the calf, with great increase of redness and heat: the least pressure or motion of the limb occasions great distress. Other patches, similar in appearance to those upon the leg and thigh, have made their appearance upon the opposite side of the body, on the right arm, and various parts of the trunk. The tongue and pulse still maintain a similar character, and the excretions continue very vitiated.—His saline medicine to be continued, with camphor mixture, and his pills, with the addition of opium. The purgative to be repeated; and the right leg was directed to be enveloped in a yeast cataplasm.

28th, nine A.M.—Has passed a most distressing night, without any quiet or lucid interval. The pulse is very quick, slightly irregular, and less forcible; occasional subsultus; tongue not materially changed; swelling, redness, and tenderness of the limb extending down to the foot, where there are some appearances of vesication, and upwards to the knee. The spots on the thigh and on the arm are increased. Dr. Magrath visited him this morning, but he refused from this period every kind of medicine.

Nine P.M.—Since our last visit he has become furious: whilst I was with him, he forced himself out of bed, where he continued more than half an hour; and, in the attempt to return him to his bed, he resisted, and made a powerful effort to pull us under the bedstead, in which he persisted; and he continued, with loud shrieking, to struggle with the united efforts of three or four strong men for a considerable time, before he sunk exhausted. The swelling of the limb is much increased; the vesications are larger and more numerous; many of them are broken, and discharge a bloody serum: the fore part of the foot is livid.

29th.—Has continued violently delirious all night: is now completely comatose, with dilated pupils, tremulous pulse, subsultus tendinum, laborious and stertorous breathing. These symptoms continued until midnight, when he expired.

*Examination of the Body, twelve hours after death.*—There were present my apprentices, and Mr. J. G. Sparke, a surgeon of Devonport, who kindly assisted me.

Skin deeply tinctured with bile over the upper parts of the trunk and face. Muscles of the face distorted, and expressive of severe bodily sufferings. Effluvia highly offensive and pungent. Extensive gangrene covering the anterior part of the right foot and leg, which was much swollen.

More than ordinary exertion required in turning back the scalp; the connecting cellular membrane between it and the pericranium having become so dense as to nearly bring them into union. Much blood effused, from numerous and larger vessels than usual being divided in performing this part of the operation. On the calvaria being forced off, the ruptured vessels in the course of the superior longitudinal sinus bled copiously. The vessels of the dura mater, and the superior longitudinal sinus, were completely gorged. On the removal of the dura mater, a large quantity of serum escaped. The cerebrum appeared as if enveloped with a stratum of coagulable lymph, the third of an inch in thickness, which, being cut into, proved to be a collection of serum, distending the tunica arachnoidea. The superficial veins of the cerebrum were loaded. Medullary substance of the cerebrum, from the surface to the corpus callosum, very dense and solid. The divided vessels, on making the different sections of it, presented extravasated spots of blood, much more expansive and numerous than ordinary. The centrum ovale greatly distended, and, on being cut into, gave exit to a large quantity of serum from the lateral ventricles, which, with the other cavities, were greatly enlarged by the contained fluid. Plexus choroides, and investing membranes throughout the ventricles, highly injected.

No appearance beneath the tentorium worthy of particular observation, excepting that the cerebellum partook of the unusual hardness which characterised the cerebrum, and that the lateral sinuses and veins were gorged with blood.

Some old pleuritic adhesions were rather extensive on the right side of the thorax.

In the abdomen, the liver, larger than ordinary, had formed adhesions to the right side; its structure more dense than natural, particularly along the anterior margin of the right lobe; its surface pallid, but its interior filled with blood. Gall-bladder distended with bile. The villous coat of the stomach was inflamed throughout, and was covered with spots of ecchymosis. The left kidney twice the natural size; its shape and structural arrangement of regular appearance. The right kidney so wasted and obscured by disease, that it could no where be felt, but was, after some search, traced (by the shrunk ureter) lying high up under the liver, and was with much difficulty removed, from the intimate connexion which it had formed, by its thickened cellular coat, to all the surrounding parts. Its organisation was destroyed; it had a schirrous feel, and was loaded with calculous matter.*

The intestines exhibited no morbid appearance, either upon their inner or outer surfaces, at the different points that were examined. Spleen, pancreas, and urinary bladder healthy. Peritoneum generally free from morbid character.

The parts beneath the cicatrix of the injured toe, from the surface to

---

* It must be superfluous to remark that this disease of the kidney was of long standing, and had no connexion with the disease under our consideration. The beautiful illustration (by the increased magnitude of the left kidney) of the power and disposition of nature to provide a remedy against the injury, which would otherwise be sustained by the system through the loss of one of these organs, is a fact sufficiently familiar to the pathologist. Quick never complained of deficiency of the urinary secretion; neither did he of vesical or urethral irritation. It may be useful to remark, that this is one among other cases that have fallen under my own observation, and that have occurred in the practice of others, wherein disease has, during life, been attributed to the liver, in consequence of the topical and constitutional evidences being exclusively of that character; and, upon dissection, the right kidney was found to be the original, or only, seat of the malady. The history of one of these cases I communicated to the Western Medical and Chirurgical Society; and, as it possesses unusual interest, I will introduce from my notes the most important features of it.

Robert Makenzie was three years and nine months old, and a healthy boy previously to this disease. A tumefaction in the right hypochondrium, and a sudden depression of strength and activity, were the first indications of it; these gradually increased until his body was perfectly extenuated, and the abdomen resembled an extensive ascites. No material pain or irritation was felt throughout, excepting what arose from the bulk of the tumor. The abdominal, thoracic, and urinary functions were regularly discharged; the excretions of the bowels being occasionally deficient of bile. The boundaries of the tumor could be plainly traced from the right hypochondrium into the opposite side of the abdomen, of a thickened and indurated feel, while the centre was elevated and elastic, as if it contained fluid. The boy died in twenty-two weeks from the commencement of his illness. The appearances upon dissection were—a large oval body, extending from the diaphragm into the pelvis, filling the whole of the right, and encroaching into the left side of the abdomen, where it pressed the whole of the small intestines into a very narrow space, the omentum being nearly obliterated by absorption. On its removal, it proved to be the kidney converted into a light-coloured pulpy substance, and weighed nine pounds (apothecaries' weight); it measured, in its greatest circumference, twenty-five inches, and in the smallest, twenty inches and a quarter. Its proper coat was in every part perfect, but increased in density. The liver was flaccid, and its substance reduced by absorption: it had a flattened tumor, in substance like that of the kidney, upon its anterior surface. The gall-bladder was nearly empty; all the other abdominal viscera of a healthy character. The right lung was covered by many dark-coloured, spongy masses, irregularly figured, and attached to the pleura by slender connexions; the three largest were shaped, and were in size, like the heart. Three flattened tubercles, containing a substance like the kidney, were situated in the parenchyma of the lungs. In the left lung there were some appearances like these last, and two large and perfect hydatids.

the bone, were perfectly free from any evidence of disease; nor did it exhibit any proofs that the bone had suffered an injury from the original accident. The cellular membrane investing the tendon of the extensor proprius, about the extremity of the second bone of the great toe, was injected with a yellow and bloody serum. This appearance did not extend in the course of the tendon, but towards the surface, and was soon involved in the general disease of the limb. The fore part of the foot, and lower part of the leg, extensively sphacelated. The cellular substance about the middle and outside of the leg, and extending down to the fascia, much swollen, and filled with bloody serum, and intersected with small cavities containing pus. The texture of the muscles between this and the bones completely destroyed, and converted into a dark-coloured mass, not unlike putrid placenta. An incision made into the cellular substance beneath the patch on the arm of the opposite side, gave exit to a quantity of greyish, unhealthy looking pus. The other spots were not opened, their appearance being precisely similar.

On the 2d of October, ten P.M., I was requested by Mr. Dryden, the assistant-surgeon of the dock-yard, who was now in charge of that establishment, in consequence of the death of the surgeon, to visit with him John Walkie, shipwright, a married man, aged thirty-eight, spare habit, sallow complexion, regular in his mode of living, and enjoying good health. Previously to my seeing the patient, Mr. Dryden informed me that Walkie had received a slight hurt upon his right leg ten days before, which had thrown off a superficial eschar, leaving a well-conditioned ulcer, that had been dressed in the usual manner with adhesive straps, on the 30th of September. In the evening of the 1st of October, he complained of uneasiness in his leg, and was soon seized with rigors, which were succeeded by heat and pain of his head. The dressings on his leg were then exchanged for a warm cataplasm, and he took aperient medicine. On the morning of the 2d (being the day on which Mr. Dryden called upon me), the symptoms continued, and there was prescribed for him calomel, and a repetition of the purgative. These operated well; but at three P.M. the pain of head and fever had increased, and he lost twenty-five ounces of blood, which was followed by a mitigation of his complaints. In the course of the evening, the activity of symptoms having recovered itself, twelve leeches were applied to the temples, and the orifices were bleeding at ten o'clock, when we visited him. He was complaining of great distress of his head, with weight of the occiput and lancinating pains through to the forehead. He had a flushed anxious countenance, and he expressed himself with fearful apprehension; he had violent throbbing of all the vessels of the head, and those of the conjunctiva were highly injected with blood. He had a sense of weight at the præcordia, which affected his respiration; and he experienced much uneasiness by pressure all around the body, from the right to the left hypochondrium; he had uneasiness in discharging his urine, and he complained of pain above the pubis. Pulse 120 and full, and the heat of the body increased; tongue was slightly coated, whitish, with an approach to brown in the centre. The ulcer upon his leg was about the size of the little-finger nail; it had a clean granulated surface, but rather pallid; there was no heat or redness around it, or in any part above it: he shrunk, however, from pressure made along the course of the absorbents, from the wound up to the inguinal glands, which were enlarged, and appeared to be the most sensitive point. Blood, which was drawn in the afternoon, exhibited slight spots of buff.

Venæsectionem repetatur ad ℥xxviii., which very sensibly reduced the forcible action of the heart and arteries, and almost totally removed the suffusion of the eyes, with decided amelioration in all the feelings of the patient. He was directed to take ten grains of calomel, and to repeat three grains every second hour; to have a purging enema injected at five in the morning. The head to be shaved, and kept cool by the application of cloths wetted in a mixture of liquor ammoniæ acetatis and spiritus vini. The body, whenever heated, to be sponged with vinegar.

3d, eight A.M.—Countenance depressed; yellowness of the skin; dullness of the eye; flushing of the right cheek; tongue inclined to dryness towards the point, and more approaching to brown; pulse very frequent and small. Head quite relieved, as well as the pain around the body at the scrobiculus cordis, and the difficulty of breathing. Complains of increased pain in the hypogastrium, which is excessively tender from the right to the left inguen, but most so in the right. There is a sense of tightness across this part, and the distress is increased upon every deep inspiration. No swelling or redness is observable in the course of the absorbents, from the ulcer to the inguinal glands, but the latter are rather more enlarged. Blood, drawn last night in a wine-glass, is covered with a thick stratum of coagulable lymph, and the crassamentum of that which was drawn in a basin is dense and heavy. A vein was again opened, and about thirty ounces drawn. During the flow the pulse gradually rose, until it became full and expansive; and it was not until this quantity was extracted, that it was deprived of a force which we considered unsafe. After this he had a very large discharge of flatus, with some dark-coloured feculent matter, being the first evacuation since the enema was injected. He said he was greatly relieved, and the improvement in his countenance corresponded with his declaration.

Four grains of opium to be taken directly; and a pill every two hours afterwards, composed of calomel, tartarised antimony, and opium. The enema to be repeated.

Eleven A.M.—Mr. Dryden visited him, and finding his pulse full and hard, with considerable return of pain, ordered twenty-four leeches to be applied to the hypogastrium.

One P.M.—Leeches have bled well. The patient says he has derived very great relief from them, and expresses himself, in consequence, with confidence of his recovery. He can bear pressure upon the groins with comparative ease. Pulse ninety, equable and soft. Blood drawn this morning cupped, and extensively buffed; crassamentum firm and tough, requiring very considerable force to break down its texture.

Eight P.M.—Has had some refreshing sleep, and still doses; has had two dark-coloured stools; says he is much easier and better in all his feelings, but has some lingering pains occasionally in the lower part of the abdomen. Countenance is pallid, but more tranquil; body covered with a warm perspiration; pulse 120, soft and regular; dryness and brown colouring of the tongue increased, (probably the effect of the opium.) —Directed a blister to be applied to the hypogastrium; the pills to be continued, and the injections repeated.

4th, eight A.M.—Mr. Dryden reported that all his symptoms were ameliorated, and his pulse 108. I was prevented from seeing him at this hour by indisposition.

Eleven A.M.—Visited him. He had enjoyed short sleeps throughout the night; his countenance was improved, and his spirits good. Tongue moist, and cleaned around the edges; pulse 120, regular and firm; has an occasional eructation, resembling hiccough, and still complains of pain above the pubis, with tightness in taking a full inspiration. Injections operated well, and the blister fully vesicated. Directed him to have twenty-four leeches applied to the blistered surface, and afterwards to be put into a warm bath. Pills and injection as before.

Nine P.M.—Leeches have sucked well, and he has been in the bath twenty minutes without the least faintness. A violent hiccough distressed him for an hour and a half before he went into the bath, but it was entirely removed by it; and, soon after being replaced in his bed, a profuse warm perspiration broke out, which still continues. Has now no pain in any part of the abdomen; can bear pressure, or full inspiration, without any uneasiness. Tongue moist, and nearly cleaned; pulse 120, expansive and regular; countenance good; mind inspired with confidence.—Directed the pills to be continued; the bleeding from the leech-bites to be arrested; and eighty drops of tincture of opium, in a draught, to be taken, if the singultus should return.

5th, seven A.M.—Was prevented from repeating my visit at this hour by indisposition. Mr. Dryden reported him to be sinking, with cold extremities, sunken pulse, anxious breathing, stools mixed with blood, senses entire.—Directed him to have warm wine, camphor mixture, with carbonate of ammonia; and the heat of the body to be sustained by bottles of warm water, &c.

Eleven A.M.—I visited him, and found him as above, with occasional discharges of dark-coloured blood from the anus. The leech-bites were still bleeding.

Twelve A.M.—Again visited him, and was informed that he had awoke out of his stupor in a state of delirium, and insisted upon getting out of bed: he exhibited so much appearance of determination, that the female attendants in the room deemed it right to call for assistance to restrain him. He had now, however, become perfectly calm, and restored to his senses. He continued undisturbed until his dissolution, at a late hour in the evening, having through the day occasional discharges of blood.

*Dissection, about twenty hours after death.*—Mr. Dryden examined the body, and reported to me the following appearances:—Slight evidences of inflammation upon the surface of the small intestines; also a patch of redness around the cardiac orifice of the stomach. Redness about the peritoneal lining of the pelvis, and effusion of some puriform fluid into that cavity. The limb exhibited no appearance of disease any where in the course of the absorbents, excepting a very small sinus about an inch above the wound, but which contained no fluid. I was prevented from being present at the dissection, in consequence of my indisposition having so increased as to render me at that time incapable of any exertion. I deeply regret that it was not thought necessary to examine the brain; and the same omission in the other cases is equally to be lamented. This most important viscus formed a point, in all the cases, on which the disease made its earliest attack, and against which it continued to direct a considerable part of its force. Quick's was the only case in which the brain was examined, and the appearances of diseased action exhibited in that organ afford ample reason for this regret in the others.

*Observations.*—All diseases arising from hurts in the dock-yard are attended by the medical officers of that establishment; consequently, the opportunities afforded to private practitioners for observing them are few. Previously to my seeing Quick, I had been informed that the disease was one of an *atonic* character; that many men had died of it; and that each had sunk in an unexpected and sudden manner, immediately after bleeding. Thus rendered cautious, I determined to commence the treatment of that with a full bleeding, and to limit my ulterior conduct of the case by such means as would guard against any sudden depression of the vital energies. The progress, termination, and dissection, afforded the strongest evidences against the truth of the opinion that the nature of the disease was asthenic, and proved, by the intensity of its effects upon the vital organs, that, to afford a chance of recovery, it was necessary to deprive the sanguiferous system of its preternatural power, with as little delay as possible.* The bleeding gave the disease the only check it received: the symptoms were all immediately subdued by it, and continued in abeyance during that and the following day; after which, the circulation recovered its tone. All the symptoms were aggravated, and continued to advance with activity and power until the fatal crisis approached. The delirium was of the ardent kind, accompanied by violent muscular exertions; and there was no decline in the strength of these or the circulation, until the patient suddenly sunk into coma on the ninth morning of his illness, in which state he remained, with stertorous breathing, until he expired at midnight. The violent pain and throbbing of the vessels of the head in the commencement, and the high cerebral irritation that existed throughout, together with the closing scene, warranted the conclusion that the immediate cause of death sprang from compression of the brain by effusion, rather than from any other cause; and the dissection corroborated that opinion.

The important facts supplied by Quick's case induced Mr. Dryden to call upon me for my opinion in the consideration of Walkie's. The history of the latter I deem of the highest importance, as it illustrates, individually, in the strongest manner, the power with which the system, labouring under one of the most violent attacks of this disease, was capable of supporting an extensive depletion, and thereby proving that the character of it could not be what is commonly understood by the term "disease of debility." It is additionally interesting in conjunction with Quick's, as it places before us, in one view, the comparative effects of the active and the less efficient treatment.

In Walkie, the symptoms were the same as Quick's, excepting that they were more intense, and that, in addition to the violent affection of the head, and viscera in the upper parts of the abdomen, the peritoneum in the lower part of the belly and pelvis was also the seat of inflammation. This man's disease became active in the evening of the 1st, and he was not bled until late in the afternoon of the next day: twenty-five ounces were then drawn from the arm, which afforded temporary

* All the bodies that were examined after death exhibited such violent effects upon the structure of vital organs, as must put the question of treatment in a decided point of view. To derive the best chance of cure in an active case, was to arrest its progress quickly. To limit the performance of this by one bleeding, with a view to economise the energies of nature for the repair of those lesions which might exist after the activity of the disease had subsided, would be providing for circumstances which, under such management, would rarely be afforded.

relief. Yet, notwithstanding this loss, and the sucking of twelve leeches from the temples, the circulation had so rallied, and the symptoms increased in the evening of the same day, that his medical attendant thought it would be compromising his safety to omit further depletion, and he was again bled, and with immediate relief of all his symptoms. On the next morning, he continued quite free from affection of the head and difficulty of breathing, with pain at the præcordia; but the pain in the hypogastrium was increased, with much tenderness upon pressure, and the pulse was quick and small. This condition of the pulse was considered to be the common result of inflammation seated in the peritoneum; and on this presumption he was again bled, and, as was anticipated, the pulse became more expansive and full as the blood flowed, until it acquired a considerable force. Notwithstanding above fifty ounces of blood had been taken from the arm, and twelve leeches sucked freely from the temples, within twenty hours, it required the further loss of thirty ounces before the circulation was again lowered sufficiently to be considered safe. The patient became rather faint, but never lost his perfect consciousness. The pain was quite removed, and he could bear pressure upon the hypogastrium with impunity. The feeling of faintness soon passed off; and the pulse, within a few hours afterwards, had resumed so much force, that Mr. Dryden (who visited him about that time) reported to me that he had, in consequence, felt some doubts upon the propriety of leaving the circulation as it then was, without further depletion.

At night, twenty-four leeches were applied to the hypogastrium, and repeated on the following evening; after which he was placed in a warm bath, and he continued in it twenty minutes, when he returned to his bed, without experiencing the least faintness or extraordinary exhaustion. His feelings were then, according to his own expressions, comfortable in every respect. The hiccough was removed; he was in a profuse, warm perspiration; and, although the pulse was quick, (no doubt accelerated by going into, and returning from, the bath,) it was soft, regular, and had sufficient force. His condition at that time was such as to create in our minds a hope that the activity of the disease was arrested, and, if it should prove so, the remaining energies of the system we thought quite ample to carry him through. But the next morning he exhibited proofs of approaching dissolution, with discharges of dark-coloured blood from the anus; and he died in the evening.

From what cause proceeded the change in the interval between ten o'clock of the night and seven of the following morning? The leech-bites had been allowed to bleed throughout the night, contrary to our directions, and we are aware of the peculiar prostrating effects of the bleeding from leeches. But it does not appear so likely that the sinking arose from that cause, as that it was connected with the discharges of blood from the anus; the result, most probably, of congestive inflammation of the liver or spleen.*

This case not only ran its course with activity and tone, but, even after the powers of life had given way, a sudden and violent action in the brain was renewed by the administration of stimuli.

Walkie was bled more extensively than any other, and he never exhibited any of those sudden and unexpected signs of debility or sinking, which are said to have characterised the other subjects, or that is peculiar to *atonic* diseases. On the contrary, he maintained a degree of power quite equal to what is ever seen, under such treatment, in phlegmonous inflammation of vital or important viscera. Upon what principle is this to be explained? Not, certainly, by the man's physical powers; for they were very unequal to many of those who had preceded him in the disease, and had sunk (it was said) from sudden debility.

His power to struggle with the disease, and the hopes which were afforded of his recovery almost to the last, must be attributed to the promptitude with which the symptoms were combated as they arose.† The fact is notorious, that, in active and important phlogistic disease, in proportion as depletion is promptly and efficiently used in conjunction with other remedies, the powers of the system are preserved; and, on the contrary, if it be resorted to with hesitation in the commencement, or if its repetition be suspended until the disease has matured itself, the effects will be similar to those which have been described in many of the fatal cases of the dock-yard.

The prime object of medical treatment is to arrest disease in its march, and, if it fail in this particular, then to moderate its violence, and conduct it to a termination the most favourable to an easy and perfect restoration of health. Compare the two cases, and it will be seen in what greater extent these objects

---

* The quantity and black appearance of the blood discharged, connected with the pain and tenderness felt around the upper part of the abdomen, difficult respiration, &c. warrant this conclusion. "In inflammation of the spleen, there is often from the rectum a discharge of black blood, from a rupture of some of the splenic vessels." "In inflammation of the liver, and congestion of that organ, we often meet with extensive hemorrhage from the anus." GOOD.—"This produces an irregularity in the portal circulation, and, gorging the cœliac and mesenteric arteries, which communicate with the hemorrhoidal vessels, large discharges of blood take place." JOHNSON.

† It is to be regretted that, in Walkie's case, the disease was allowed to advance from the evening of the 1st until the afternoon of the next day, before the depletion was used. It is fair to presume, from the whole of the history of that case, that, had the active means which were afterwards pursued been adopted at the commencement, the result might have been different.

were attained in Walkie than in Quick. Compare the progress of symptoms in each, and also the contrast of appearances upon dissection, and it will demonstrate how greatly the active antiphlogistic treatment preponderated over the passive.

Upon these grounds, I dissent from opinions that have been expressed,* and draw the following conclusions:—

1. The two cases detailed in this paper are genuine specimens of the fatal disease which occurred at Devonport, in the autumn of last year, and which has been commonly called Dock-yard disease.

2. The symptoms attending the progress and consummation of the disease, together with the dissections, prove that it possessed a tonic and active character; and that its destructive influence was chiefly exerted through the medium of the sanguiferous system.

3. That all those means comprehended in the general term *antiphlogistic*, regulated with promptitude and efficiency, proportionate to the activity and intensity of the symptoms, formed the treatment best calculated to relieve.

It may, however, be objected to these inferences, that the large proportion of deaths, which are exhibited in the history that has been published, furnishes a fact in opposition to them, and proves that a tonic and stimulant plan of treatment should supersede the antiphlogistic. I would answer, that, to support such an opinion, in opposition to the evidence supplied by this paper, it is not sufficient merely to state that twelve men, who died of this disease, had all been bled, and therefore that bleeding must be improper; but it is necessary that the history of each case should be minutely and progressively stated, so as to exhibit the real condition of the patients, linked with the treatment they respectively received. The effect of this would be to prove in what extent the antiphlogistic treatment had been satisfactorily used in all those men who died. It would also afford an opportunity of observing what resemblance (in degree as well as kind) there was between these and the case that is represented to have been cured by the opposite mode of treatment.

The cases which have been published do not supply this *necessary sort of evidence*, and their brevity is accounted for in the Preface: it is there stated that no notes were originally taken with a view to publication; but that the relatives and friends of the deceased had been called upon to render them as accurate as possible under these circumstances; so that there is not only deficiency of matter in the relation of these cases, but consider-

---

* See *Remarks on Irritative Fever, commonly called Dock-yard Disease, &c.* by Dr. Butter.

able objection to the mode by which it was obtained. These facts deprive them of importance, and must render nugatory any attempt to convert them into a precedent for the establishment of any important point of practice.

The subject of inquiry is not one of idle curiosity. In all scientific investigations which involve the life or happiness of man, it is essential to ascertain that the general principles which are to influence our future conduct are just and true. In medical reasoning, it does not follow, because a certain practice was unsuccessful, that therefore it was wrong: the result might arise from the ungovernable nature of the malady. The symptoms of high excitement which characterised the cases that I have detailed, from their commencement to their termination,—the evidences of inflammatory action upon the internal structures and surfaces of the body,—speak a language which cannot be misunderstood; and, if bleeding and other antiphlogistic means are not to be pursued under such circumstances, it is most important to determine the line which is to define our practice.

*Case of Ann Scobell.*

I subjoin a case that occurred in my practice during the last fortnight, and which may be considered important, connected with the subject which we have been considering.

Ann Scobell, a poor woman, aged about fifty, sallow complexion, and spare habit of body, while employed about ordinary household work, was suddenly seized with rigors and great loss of power, about seven o'clock of the evening of the 16th instant. It was soon succeeded by a pungent pain, which fixed itself upon the outside of the right forearm. This shortly afterwards extended to the axilla, and down to the fingers, and so increased in violence that she was incapable of moving the limb, or suffering it to be touched. At ten the same evening I visited her: she then described the pain as pervading the whole of the right side of the body, from the head to the feet. Her skin was much heated; her pulse was 100, full and strong; her tongue slightly coated. She laboured under much mental agitation, from apprehension of danger. There was considerable distress of the head, and pulsation of the arteries; a difficulty of breathing, and pain with tenderness in the right side; a slight blush of red appeared upon the back of the hand. Not the slightest topical injury could be detected any where.

Bled her to thirty ounces, which produced an approach to faintness, that continued for many hours. Was immediately relieved of all her pains; could move her limb with freedom in every direction. Directed her to take a pill every four hours, with calomel, tartarised antimony, and opium; and a purgative draught, with salts and infusion of senna, directly.

17th.—Head and side are quite relieved. Considers herself better in every respect. Pulse is ninety, and softer; temperature of skin is diminished; tongue is rather more coated, and inclined to brown and dryness; has had no stools, nor discharge of urine. Arm continues free from pain, and she can move it in any way with impunity; but, from the fingers to the shoulder, the limb is covered with vivid red lines.—Directed the pills to be continued, without the opium; and the purgative to be repeated, with the addition of ten grains of jalap.

Evening of 17th.—Red streaks of arm are fading; tongue is moister and cleaner; pulse still diminishing in frequency and force. Purgative has acted well, and there has been a copious discharge of urine. Excretions from the bowels vitiated.—Directed a repetition of the purgative in the morning.

18th.—Complains of fixed pain at the scrobiculus cordis; also some pain and swelling in the middle joint of the ring-finger of the affected arm: every other species of pain is removed. Pulse, tongue, and temperature of the body, are natural. Medicines have again acted well.—Directed twelve leeches to the stomach, and the medicines to be continued, with a cataplasm to the finger.

19th.—Some slight pain in the stomach and in the finger constitute the whole of the disease which remains. There is not the least appearance of constitutional disturbance.—Directed the pills to be continued, with an addition of opium.

19th.—Pain at scrobiculus cordis is wholly removed; but the pain and swelling about the joint of the finger is increased.

23d.—The state of things as last described has continued, with a slight increase of swelling of the finger, where a fluctuation is now evident, which was opened, and discharged a small quantity of pus.

28th.—Convalescent.

